# Improving quality of malaria treatment services: assessing inequities in consumers' perceptions and providers' behaviour in Nigeria

**DOI:** 10.1186/1475-9276-9-22

**Published:** 2010-10-11

**Authors:** Obinna Onwujekwe, Eric Obikeze, Benjamin Uzochukwu, Ijeoma Okoronkwo, Ogochukwu C Onwujekwe

**Affiliations:** 1 Department of Health Administration and Management, College of Medicine, University of Nigeria, Enugu, Nigeria; 2 Health Policy Research Group, Department of Pharmacology and Therapeutics, College of Medicine, University of Nigeria, Enugu, Nigeria; 3 Department of Community Medicine, College of Medicine, University of Nigeria, Enugu, Nigeria; 4 Department of Nursing Sciences, College of Medicine, University of Nigeria, Enugu, Nigeria; 5 Department of Pharmacy, University of Nigeria Teaching Hospital, Ituku-Ozalla, Enugu, Nigeria

## Abstract

**Background:**

Information about quality of malaria treatment services of different healthcare providers is needed to know how to improve the treatment of malaria since inappropriate service provision leads to increased burden of malaria. Hence, the study determined the technical and perceived quality of malaria treatment services of different types of providers in three urban and three rural areas in southeast Nigeria.

**Methods:**

Questionnaire was used to interview randomly selected healthcare providers about the technical quality of their malaria treatment services. Exit polls were used to obtain information about perceived quality from consumers. A socio-economic status (SES) index and comparison of data between urban and rural areas was used to examine socio-economic status and geographic differences in quality of services.

**Results:**

The lowest technical quality of services was found from patent medicine dealers. Conversely, public and private hospitals as well as primary healthcare centres had the highest quality of services. Householders were least satisfied with quality of services of patent medicine dealers and pharmacy shops and were mostly satisfied with services rendered by public and private hospitals. The urbanites were more satisfied with the overall quality of services than the rural dwellers.

**Conclusion:**

These findings provide areas for interventions to equitably improve the quality of malaria treatment services, especially for patent medicine dealers and pharmacy shops, that are two of the most common providers of malaria treatment especially with the current change of first line drugs from the relatively inexpensive drugs to the expensive artemisinin-based combination therapy, so as to decrease inappropriate drug prescribing, use, costs and resistance to artemisinin-based combination therapy.

## Introduction

Malaria is a major public health problem in Nigeria and its treatment is sought from a broad spectrum of public and private healthcare providers [1,2]. The erosion of the public health system, arising from mismanagement, has contributed to the growth of the private sector and, in particular, the rise in the "informal" private sector as a source of treatment [1]. Patients often resort to the unregulated private commercial sector, where treatment may be inappropriate but at a lower cost [1]. The informal private sector is now a major source of anti-malarial drugs, but these providers, especially patent medicine dealers (patent medicine vendors) generally provide low quality services [3]. A study of treatment of childhood malaria in Zambia found that, in most cases, drugs were bought at pharmacies or local shops, but these treatments were often inconsistent with national treatment guidelines; for example, they may include counterfeit drugs, drugs of poor quality, incorrect dosing and irrational prescription practices [4]. Private sellers such as patent medicine dealers often lack knowledge of appropriate treatment and are influenced by advertising and profit motives [5].

Perceived and actual quality of care administered at all levels of health care are major determinants of health outcomes and consumer's choice of treatment provider [6,7]. In many places, health services from both public and private providers are of questionable quality, with long waiting times, inaccurate diagnosis, inappropriate prescription and advice and frequent drug stock-outs. The use of presumptive malaria diagnosis without laboratory support, which is a common diagnostic procedure for malaria in Nigeria and in many sub-Saharan Africa (SSA) countries in both public and private facilities predisposes to poor quality of malaria diagnosis and treatment. Case history has proven an unreliable diagnostic method; in one instance it was found that nurses had a 10% accuracy of malaria diagnosis using case history compared to doctors with a higher level of accuracy [8].

Information about quality differentials across providers is needed to identify the loci for intervention, as poor quality of healthcare services is a major contributor to the high direct and indirect costs to patients [6,9]. Studies from a number of countries note that the technical capacity of private clinics is perceived as inferior [1,8]. If a patient is very ill, the public sector may be preferred for its sophisticated equipment and wide range of staff [10]. Patients may also perceive private providers to rely excessively on diagnostic tests and charge very high prices and they are skeptical about the motivation of private providers, believing they are more interested in generating income for themselves than in the welfare of their patients [10,11].

Public treatment services are themselves frequently inefficient, of poor quality, and underutilized and often lack drugs and diagnostic facilities [1,12]. Inappropriate prescription is common in these facilities [13], reducing the quality of care, wasting resources and potentially contributing to the spread of drug resistance [14]. The poor quality of care at public facilities, crowds, long waiting times and cursory consultations, was a key factor in the preference for private providers [6]. Other studies looking at a broader range of diseases in Nigeria found widespread inappropriate drug use, low quality of treatment, and ineffective regulation [12,15-18].

There is paucity of documented evidence about actual quality of malaria treatment provided by different private and public providers. The exploration of providers used and quality of malaria treatment services they provide is needed to identify and correct problems associated with quality of malaria treatment services [7]. Also, there is paucity of knowledge especially within the private sector, particularly whether the poor are more likely to use some private providers that deliver poor services [9]. Poor quality of services deter use of health services, particularly by the poor [6]. Some researchers showed that there were positive associations between socio-economic status and health seeking from an appropriate provider for fever [19].

This paper provides information on the differential quality of perceived and providers' stated quality of malaria treatment services from a broad spectrum of public and private providers. Hence, the paper assesses the relative quality of malaria treatment services offered by different types of healthcare providers. The paper also shows whether there are socio-economic and geographic differences in quality of services rendered to different consumer groups as well as influences on perceived quality of treatment. The information generated by this study will help design policy measures to strengthen the treatment component of the malaria control strategy, especially with the use of the expensive artemisinin-based combination therapy (ACT) as first-line treatment for malaria.

## Methods

### Study design

It was a cross-sectional study. Data was collected using provider interviews and exit polls through questionnaires. The study site was Anambra State, southeast Nigeria. The state has a high malaria transmission rate throughout the year. Six communities were chosen for the study and each site area had a full complement of providers. Awka, Nnewi and Onitsha were selected based on being urban. Njikoka, Aguata and Ogbaru were selected based on their being rural. The communities were randomly selected using two-stage sampling, by first stratifying the communities according to whether they have a public hospital and then randomly selecting the sites from those that have public hospitals.

#### Provider interview

The sampling frame included the major types of private and public providers that use bio-medical (orthodox) drugs to treat patients and at all levels of care in public and private facilities. Private providers were hospitalspharmacy shops (PS), laboratories and patent medicine dealers (PMDs). Laboratories were included because in real practice, they actually provide malaria treatment practices instead of just diagnostic services that they are permitted to provide. Public providers were hospitals and Primary healthcare centres (PHCs). The sample size was determined by considerations of the range of providers and feasibility. There was listing of providers in the study areas using their association registers. Snow ball approach was used to reach the providers who were not included in the association register. Proportionate allocation was used to choose different numbers of providers from the urban and rural areas because the urban areas have more population of respondents. A total of 50 public and private providers in each urban and 25 in each rural area were selected, adding up to 225 providers The sample size and breakdown of providers was determined based on their utilisation rate by consumers using existing data [20]. The sampling of private providers was hence made with probability proportionate to size. All public providers in each study area were included in the study because they were not many.

A structured questionnaire was administered by trained field workers to the heads or owners of selected public and private providers/outlets. The providers were drawn from PMDs, PHC centres, public and private hospitals as well as laboratories. The employees running the facilities were interviewed in the absence of the owner or head. In the case of hospitals, the providers that were interviewed were medical doctors, whilst they were pharmacists and laboratory technicians in the case of pharmacies and laboratories respectively. In the PHC centres, either the head or the next highest available ranked healthcare provider was interviewed. Finally, in PMDs, the owners or the highest ranked assistant was interviewed. Information was obtained from the provider about how they diagnosed and treated malaria with artemisinin-based drugs, especially ACTs. The selected providers on the aggregate treated more than 85% of adult malaria and more than 90% of childhood malaria [2].

#### Exit polls

In order to obtain accurate data, at least ten clients from each of the providers were interviewed just after receiving treatment from each of the 225 providers, giving a minimum sample size of 2,250 exit polls. However, when the number of clients interviewed were aggregated across the different groups of providers, the numbers were 860 in PMDs, 273 in PHC centres, 195 in public hospitals, 716 in private hospitals, 222 in pharmacy shops and 38 in laboratories, which were adequate for making statistical inferences because the computed overall minimum sample size for respondents was achieved. The respondents on immediate exit from the providers' premises were asked whether or not the quality of services that they just received based on five attributes of treatment were acceptable or not acceptable to them. The acceptability was based on consumers' self-assessed perception of the quality of diagnosis and other dimensions of quality. The dimensions were: Diagnosis; Correct drug; Correct dosage; Instructions on how to take the drugs; and Follow-up information on what to do if they did not recover. Data was also collected on specific diagnostic actions which included provision of blood tests, taking case history, measuring of temperature, heart rate and respiratory rate. In case that the provider did not undertake any specific diagnostic action before prescribing treatment of dispensing drugs, this was recorded as "no diagnosis". Data was also collected on the specific type of main healthcare provider (doctors, nurses, medical assistants, pharmacists, community-health workers and PMDs that treated the patients) Data was also collected household socio-economic status (assessed through ownership of assets and household expenditure).

Trained data collectors interviewed patients or their carers (in case of children) that had just received treatment for fever from each of the sampled providers using a pre-tested semi-structured questionnaire (after the providers had been interviewed). Inclusion criterion was all patients who were accepted through diagnosis to have symptoms of malaria and such patients were referred to the data collector by the provider for interview after treatment had been given. Those who did not have malaria symptoms, and were not referred to the data collector from the provider were excluded from the exit poll. The first ten eligible respondents were selected and interviewed as they exited the providers' facilities.

##### Ethical approval and Consent

The study was approved by the Ethics Committee, University of Nigeria. Informed consent was obtained from the respondent who read and agreed to be interviewed having accepted the conditions.

## Data Analysis

### Provider behaviour

Tabulations were used to examine variables for stated quality elicited from the various providers in the different areas. The variables on providers' stated quality between the different types of providers were compared. The variables were: method of diagnosis of malaria; and provision of ACTs and other anti-malarial drugs. This was based on the premise that appropriate treatment consists of proper diagnosis and treatment with nationally recommended drugs. Other variables that were explored included the type of healthcare provider that provided treatment, the actions taken for diagnosis (blood tests, history taking, measurement of temperature, heart rate and respiratory rate).

### Perceived quality

Tabulations were used to examine variables for perceived quality elicited in the exit poll. These variables were the consumers' perceptions of the quality of diagnosis, drugs that were provided, specified dosage, follow-up instructions and general information provided to the patients. They were all measured as binary variables (1 = acceptable and 0 = not acceptable).

Cross-tabulations and logistic multiple regression analyses were used to examine the statistical relationship of independent variables with perceptions about the five perceived quality attributes. In cross-tabulation the binary place of residence variable and SES quartiles were tabulated against the five consumer perceived quality attributes respectively. The five different dependent variables were perceptions of the quality of diagnosis, drugs given, specified dosage, follow-up instructions and general information provided to the patients. Cross tabulations were also used to analyze relationship of specific diagnostic actions with different healthcare providers.

In logistic regression analysis, the independent variables were the place of residence (urban = 1; rural = 2), age (years), sex (male, female) and maximum educational level of schooling that the respondent attained, as well as SES weight. The base variable for educational status was 'no formal' education. The dependent variables were the five consumer perceived quality attributes that were described in the preceding paragraph. The statistical significance and the percentage of correct predictions of the logistic models were examined. Statistical significance of the independent variables was set at the 5% level.

### Equity analysis

Equity analysis examined the relationship between geographic location and socio-economic status (SES) with the key quality variables. A continuous asset-based socio-economic status (SES) index [2] was generated using principal components analysis (PCA) with information from the households' asset holdings together with the households' per capita weekly cost of food. The assets considered were households' ownership of motorcar, motorcycle, radio, refrigerator, television set and bicycle. Weights for the SES index where derived using the first principal component of the PCA. One SES model was estimated for pooled data of urban and rural areas so that households could be directly compared. The SES index was disaggregated into quartiles ranging from Quartile 1 (Q1) which was the most poor group and Q4, the least poor group. The SES index was used to examine whether there were systematic differences in quality by SES. The ratio of the lowest SES to the highest SES was computed as the measure of inequity. A binary variable was created to represent urban and rural residence.

#### Definitions

***Providers' stated quality ***refers to the effectiveness of care in producing achievable health gain. It reflects the stated appropriateness and technical competence of services provided.

***Perceived quality ***refers to quality as assessed from the patient's perspective.

## Results

### General characteristics of the consumers

The majority of respondents were females, married people and those mostly in their mid-thirties were the majority of respondents (Table 1). The table also shows that most of the respondents had some formal education and spent an average of 11.5 years in school. Whilst 64.5% of visits to healthcare providers were by the respondents, the rest were for other household members including children.

**Table 1:** Socio-economic and demographic characteristics of the respondents from the exit poll

	Enugwu-Ukwu n = 246	Awka n = 498	Ekwulobia n = 294	Nnewi n = 495	Onitsha n = 498	Okpoko n = 275	Urban n = 1488	Rural n = 818	Combined n = 2306
Females: n (%)	139(56.5)	271(54.4)	173(58.8)	303(61.2)	350(70.3)	201(73.0)	924(62.1)	514(62.8)	1437(62.3)

Age: mean (SD)	37.4 (13.37)	37.02 (13.17)	36.7 (11.61)	35.37 (9.45)	35.94 (10.93)	33.09 (7.31)	36.37 (11.56)	37.21 (12.98)	36.05 (12.05)

Attended school: n (%)	221(89.8)	462(92.8)	253(86.1)	485(98.0)	487(97.8)	250(90.9)	1434(96.4)	724(88.5)	2158(93.6)

No of years of education: mean (SD)	11.17 (9.10)	11.97 (4.20)	10.91 (3.88)	10.80 (3.59)	11.79 (3.4)	10.87 (3.47)	11.60 (4.68)	11.13 (5.68)	11.53 (3.39)

Whether married: n (%)	144(58.5)	239(50.0)	173(58.8)	380(76.7)	350(70.3)	214 (77.8)	969 (65.1)	452 (55.3)	1421(61.6)

### General characteristics of the providers

Seven major providers of malaria treatment services were accessed for the study: public hospitals, private hospitals, PHC centres, pharmacy shops, maternity homes, PMDs and laboratories. There were 137 PMDs, 4 mixed goods sellers, 22 PHC centres, 6 maternity homes, 20 private hospitals, 3 other low level providers such as community-health workers and itinerant drug sellers, 8 laboratories, 11 pharmacy shops and 11 public hospitals. Three other high level providers were interviewed. It was found that 60.4% of the respondents were the heads of the facilities and the rest were their representatives. The average numbers of years of formal education that PMDs, providers in public hospital, pharmacy shops, laboratories, PHC centres and providers in private hospitals were 5.0 (SD 2.9), 19.2 (SD24.0), 15.0 (SD 4.8), 17.4 (SD 3.1), 15.0 (SD 2.6) and 19.5 (SD 4.9) respectively.

The average number of clients that the PMDs, public hospitals, pharmacy shops, laboratories, PHC centres and private hospitals were attended to in their last business days prior to the interview were 12.2 (SD 10.2), 19.1 (SD 6.9), 24.7 (SD 28.0), 14.1 (SD 15.4), 9.3 (SD 3.5) and 17.5 (SD 18.1) people respectively.

#### Diagnosis of malaria and provision of Artemisinin-based drugs

History taking was used in the diagnosis in 2098 (90.4%), blood test in 155 (19.6%) and others in 14 (5.8%) of the cases (Table 2). However, most of the providers combined the different diagnostic methods. In a number of cases, especially in public and private hospitals, and laboratories, history taking was a major additional diagnostic method. The least use of blood tests was in primary healthcare (PHC) centres, whilst blood tests were most highly used in laboratories. However, both public and private hospitals used blood tests in 42% of cases to diagnose malaria (Table 2). The chi-square of association of different providers with all the different diagnostic actions were statistically significant. The differential provision of artemisinin-based drugs by different providers is shown in Table 3. Artemisinin-monotherapy (AMT) was more prescribed and procured compared to artemisinin-based combination therapy (ACT). However, higher proportions of ACTs were prescribed in public hospitals, compared to private hospitals and other providers (Table 3).

**Table 2:** Method of diagnosis of malaria by different healthcare providers

	Patent Medicine Dealer n = 860 n (%)	PHC centre n = 273 n (%)	Public Hospital n = 195 n (%)	Pharmacy n = 222 n (%)	Private hospital n = 716 n (%)	Laboratory n = 38 n (%)	Chi-square (p-value)
Blood test	24 (2.79)	10 (3.7)	86(41.9)	2 (0.9)	297 (41.5)	31 (81.5)	621.8 (0.0001)
History	725(84.3)	271(99.3)	190(97.4)	203 (91.4)	664 (92.7)	29 (76.3)	87.7 (.0001)
Temperature	118(13.7)	230(84.2)	89(45.6)	46 (20.7)	465 (64.9)	3 (7.8)	696.5 (.0001)
Heart rate	68(7.9)	134(49.0)	72(36.9)	27(12.1)	346 (48.3)	1 (2.6)	430.5 (.0001)
Respiration	53(6.1)	116(42.5)	75(38.5)	10(4.5)	323 (45.1)	1 (2.6)	447.1 (.0001)
No diagnosis	119(13.8)	20(7.3)	4(2.1)	12 (5.4)	24 (3.4)	1 (2.6)	79.6 (.0001)
Others	36(4.1)	9(3.2)	16(8.2)	4(1.8)	67 (9.3)	0 (0)	33.6 (.0001)

**Table 3:** Differential provision of artemisinin-based drugs by different healthcare providers

	Patent Medicine Dealer n = 860 n (%)	PHC centre n = 273 n (%)	Public Hospital n = 195 n (%)	Pharmacy n = 222 n (%)	Private hospital n = 716 n (%)	Laboratory n = 38 n (%)	Chi-square (p-value)
**Drugs prescribed**							
AS monotherapy	43 (5.2)	6(2.2)	5 (2.6)	27(7.5)	84 (6.1)	2 (5.2)	45.2 (.0001)
ACT	5 (0.6)	1(0.4)	15(7.7)	7 (3.2)	10 (1.4)	0 (0)	56.4 (.0001)

**Drugs procured**							
AS monotherapy	44 (5.2)	5(1.8)	16 (8.2)	26 (11.7)	76 (10.6)	0 (0)	58.3 (.0001)
ACT	8 (0.9)	0(0)	15 (7.7)	9 (4.1)	10 (1.4)	1 (2.6)	36.4 (.0001)

#### Consumers perceived quality of treatment services by types of healthcare providers from exit poll

The quality of treatment from different providers differed depending on the indicator of quality assessed (Table 4). As would be expected, the quality of laboratory diagnosis was perceived to be best in laboratories followed by public and private hospitals, whilst the lowest perceived diagnostic quality was in pharmacies. The laboratories had the lowest perceived quality scores for all other indicators. The public hospibtals, private hospitals and PHC centres were generally the best performing. Patent medicine sellers had fairly acceptable levels of quality on all the five indicators. In scoring the two common and very important quality attributes, which were (availability and quality of drugs), pharmacies closely followed by private hospitals ranked highest in both attributes. The patent medicine dealers were ranked lowest in both availability and quality of drugs.

**Table 4:** Consumers' perceived quality of treatment services offered by different healthcare providers from exit poll

	Diagnosis (%)	Drug given (%)	Correct dosage (%)	Instructions (%)	Information (%)
Laboratory	100	41.6	47.2	44.4	19.4
Pharmacy	49.7	89.5	80.5	82.9	52.1
Patent Med Dealer	56.1	88.6	81.0	84.9	51.3
PHC centre	77.3	91.2	85.3	87.1	46.8
Private hospital	84.4	91.1	85.9	88.0	64.9
Public hospital	85.6	91.8	79.5	80.0	58.9
Chi-2 (p-value)	131.3 (p < .0001)	1.6 (p = .20)	43.7 (p < .0001)	61.1 (p < .0001)	75.3 (p < .0001)

#### SES and R/U differences in diagnosis

Table 5 shows that the two better-off SES had more blood tests and more history of the illness when compared with the two worse-off SES groups. The better-off SES groups also had more of their temperature and respiratory rates measured. However, more of the counting of the pulse rate was with the most-poor SES group. The urbanites in general also had better diagnosis on all points than the rural dwellers.

**Table 5:** SES and Rural-Urban differences in methods of diagnosis of malaria

	Blood tests	History	Temperature	Heart rate	Respiration n (%)
	n (%)	n (%)	n (%)	n (%)	
**SES**					
Q1 most poor	100 (18.3)	469 (85.9)	185 (33.9)	65 (11.9)	123 (22.5)
Q2 very poor	95 (17.4)	486 (89.2)	226 (41.5)	43 (7.9)	140 (25.7)
Q3 poor	110 (20.1)	515 (94.2)	261 (47.7)	36 (6.6	197 (36.0)
Q4 least poor	128 (23.5)	506 (92.8)	241 (44.2)	35 (6.4)	171 (31.4)
Equity (Q1:Q4) ratio	0.8	0.9	0.8	1.9	0.7
Chi-square(p-value)	7.4 (.061)	26.6 (< .0001)	23.3 (< .0001)	14.2 (.003)	28.6(< .0001)
**R/U differences**					
Rural	203 (24.7)	757 (92.2)	331 (40.3)	213 (25.9)	171(20.8)
Urban	252 (16.8)	1340 (89.4)	624 (41.6)	447 (29.8)	419 (28.0)
Equity (R:U ratio	1.47	1.03	0.96	0.87	0.74
Chi-square(p-value)	21.1 (< 001)	4.8 (.028)	0.38 (.54)	3.9 (.048)	14.3 (< .001)

#### SES and R/U differences in prescribers of treatment

Table 6 shows that more qualified health personnel such as doctors and pharmacists prescribed treatment for the better-off SES groups and for urbanites (p < 0.05). Conversely, patent medicine dealers prescribed more of the treatment to worse-off SEs groups and to rural dwellers (p < 0.05).

**Table 6:** Rural-Urban differences in specific provider that prescribed treatment of malaria

	Doctor	Nurse	Medical assistant	Pharmacist	Community Health Worker	Patent Medicine Dealer
**Exit poll**						
Q1 most poor	134	72	18	22	12	189
Q2 very poor	140	86	15	37	23	165
Q3 poor	174	82	26	50	23	145
Q4 least poor	172	90	19	57	25	134
Equity (Q1:Q4) ratio	0.78	0.8	0.95	0.39	0.48	1.4
Chi-square(p-value))	11.8 (.001)	2.0 (.16)	.57 (.45)	18.9 (.001)	3.9 (.047)	17.0 (.001)
**R/U differences**						
Rural	279	92	11	38	32	328
Urban	589	142	19	138	69	520
Equity (R:U ratio)	0.87	1.18	1.0	0.5	0.85	1.15
Chi-square(p-value)	6.5 (.011)	1.7 (.19)	0.02(.88)	15.9 (< .001)	0.63 (.42)	6.3 (.012)

#### SES and R/U differences in perceived quality of services

The better-off SES groups perceived higher quality of services compared to the worse-off SES groups (Table 7). This was especially in terms of quality of diagnosis, drug given correct dosage of the drugs, instructions about how to take the drugs and general health information. The rural-urban differences in perceived quality of treatment were mixed. There were no statistically significant differences in diagnosis, drugs given and correct dosage. However, whilst the urbanites perceived higher quality of follow-up instructions about the treatment, the rural dwellers perceived higher quality in general health information given. The rural dwellers were generally more satisfied with the quality of services that they received compared to the urbanites (Table 8).

**Table 7:** SES and Rural-Urban differences in perceived quality of treatment services by types of healthcare providers

	Diagnosis n n (%)	Drug Given n (%)	Correct Dosage n n (%)	Instructions n (%)	Information n (%)
**SES**					
Q1 most poor	326 (59.7)	474 (86.8)	409 (75.1)	423 (77.5)	240 (44.0)
Q2 very poor	376 (68.9)	469 (85.9)	440 (80.7)	458 (83.4)	279 (51.2)
Q3 poor	421 (77.1)	511 (93.6)	482 (88.3)	494 (90.5)	360 (66.1)
Q4 least poor Equity (Q1:Q4) ratio	405 (74.3) 0.8	494 (90.6) 0.95	461 (84.6) 0.9	480 (88.1) 0.9	329 (60.4) 0.7
Chi-square(p-value)	48.8 (< .001)	24.0 (.001)	38.6 (< .001)	44.6 (< .001)	65.7 (< .001)

**R/U differences**					
Rural	581 (70.7)	733 (89.2)	666 (81.2)	677 (82.4)	508 (61.9)
Urban	1052 (70.3)	1340 (89.5)	1246 (83.2)	1295 (86.5)	783 (52.3)
Equity (R:U) ratio	1.01	1.00	0.98	0.95	1.18
Chi-square (p-value)	1.88 (.39)	1.9 (.40)	3.2 (.21)	8.6 (.013)	21.5 (< .001)

**Table 8:** R/U differences in general level of satisfaction with services

	Very satisfactory n (%)	Satisfactory n (%)	Not satisfactory n (%)	Do not know n (%)
**R/U differences**				
Rural	509 (62.0)	300 (36.0)	2 (0.01)	8 (1.0)
Urban	817 (56.0)	583 (40.0)	38 (3.0)	38 (3.0)
Equity (R:U) ratio	1.11	0.90	0	0.33
Chi-square (p-value)	(.003)	(.053)	(.002)	(.004)

### Multiple regression analyses

Logistic regression analysis showed that perceived quality of diagnosis was positively and statistically significantly related to place of residence (p < 0.01), and SES (p < 0.01). It was however negatively and statistically significantly related to whether the respondents' highest education level was primary school (p < 0.05), junior secondary school (p < 0.05), senior secondary school (p < 0.05) and university (p < 0.01). Place of residence (p < 0.01) and SES (p < 0.01) were the only variables that were positively and statistically significantly related to perceptions of quality of drug. SES (p < 0.01) was the only variable that was statistically significantly related to perceived quality dosage and follow-up instructions and the relationship between the two sets of variables was positive. Finally, perceived quality of general information provided was positively and statistically significantly related to age of the respondent (p < .05) and SES (p < 0.01). All five logistic regression models were statistically significant (p < .01) and all predicted more than 70% of the observations.

## Discussion and Recommendations

The services from the highly used drug sellers, patent medicine dealers (PMD) and pharmacy shops (PS) were of lowest quality. These findings are similar to those of other studies that show that medicine sellers offer a service to patients that is widely used but generally of poor quality [21]. Public hospitals, private hospitals and PHC centres had the best levels of both perceived and technical quality of services, although not generally optimal in all the quality indicators used in the study. A similar study found mostly erroneous drug prescribing practices among private shops, indicating the need for innovative and effective approaches to achieve rational prescribing practices [22].

As was found in this study and by other studies, a range of problems had been identified with the quality of malaria treatment, both in formal health facilities and by more informal providers of care such as shopkeepers [1]. Some of these problems are related to diagnosis [1]. In this study, less than half of public and private hospitals used blood tests to diagnose malaria and the tests were rarely used by patent medicine dealers that provide the bulk of malaria treatment services. There were few incidences of prescription of ACTs. One of the few previous studies that examined the quality of treatment of health providers found that patent medicine dealers provide low quality services to their clients [8]. A small study in rural Nigeria identified inappropriate dispensing of anti-malarials by the majority of patent medicine dealers [23].

The study would have been enriched if qualitative research methods such as focus groups discussions (FGDs) and indepth interviews were used to further explore issues of quality of treatment from both providers' and consumers' perspectives. This lack of both FGDs and IDIs is a study limitation, which future studies in this research area should incorporate when collecting data so that information on differential quality of treatment will be more robust. Also a bias that could have been introduced to the study was the fact that data was collected from patients that were referred to the data collector by the provider for interview after treatment has been given. Hence, providers may have referred only patients where they felt that they provided good quality of treatment services.

There were inequities in the quality of malaria treatment services because the better-off SES and urbanites were treated by more qualified personnel, had better diagnostic procedures and instructions from the providers compared to worse-off SES and rural dwellers. This was buttressed by the results of logistic analysis that showed a consistent positive association of SES with all perceived quality attributes. However, in two of the logistic models, rural residence was positively associated with perceived quality of care. The rural dwellers possibly perceived higher quality of treatment because they may have lower quality expectations and so were satisfied with the treatment that they received. Some authors showed that poor people bear a disproportionate burden of the disease and have poor health-seeking behaviour, thus leading them to seek treatment from "low-level" providers and avoid any from of laboratory-based formal diagnosis [1,2,7,24-27], which consequently increases the burden of the disease on them [28]. The fact that the two better-off SES groups perceived higher quality of treatment than the worse-off SES was not surprising considering that they received higher quality diagnosis and were treated more by more qualified personnel.

The finding that people were least satisfied with the services, especially diagnostic procedure and follow-up information, at PMD and PS, the two most common sources of treatment of malaria is an area for policy and programme interventions especially with the current change of first line drugs from the relatively inexpensive chloroquine and SP to the expensive ACT so as to decrease inappropriate drug prescribing, use, costs and resistance to ACT.

The current change of first line drugs to expensive ACT implies an urgent need to improve quality of treatment in both public and private sectors. Given that majority of low-level providers, especially patent medicine dealers, are not trained health professionals, the low quality of treatment found calls for interventions to enhance accurate diagnosis at first attempt and facilitate the use of appropriate drug by retailers and to reduce overall treatment cost and possible incidence of drug resistance [29]. Also, interventions are needed to improve quality of follow-up information in PMD, PS and PHC centres so as to decrease inappropriate drug prescribing, use, costs and resistance to ACT. The interventions could include training, providing of job aides and closer supervision by malaria control managers.

## Competing interests

The authors declare that they have no competing interests.

## Authors' contributions

OO conceived the study, participated in the design and performed statistical analysis. BU and EO participated in the design of the study and coordination. IO and OCO participated in literature review and data collection. OO drafted the manuscript. All authors read and approved the final manuscript
